# Early decision-analytic modeling – a case study on vascular closure devices

**DOI:** 10.1186/s12913-015-1118-3

**Published:** 2015-10-27

**Authors:** Alina Brandes, Moritz F. Sinner, Stefan Kääb, Wolf H. Rogowski

**Affiliations:** Helmholtz Zentrum München, German Research Center for Environmental Health (GmbH), Institute of Health Economics and Health Care Management, Ingolstädter Landstrasse 1, 85764 Neuherberg, Germany; Department of Medicine I, University Hospital Munich, Ludwig-Maximilian University, Marchioninistrasse 15, 81377 Munich, Germany; Deutsches Zentrum für Herz-Kreislauf-Forschung (DZHK, German Centre for Cardiovascular Research), partner site Munich Heart Alliance, Biedersteiner Strasse 29, 80802 Munich, Germany; Ludwig-Maximilian University, Munich, Institute and Outpatient Clinic for Occupational, Social and Environmental Medicine, Clinical Center, Ziemssenstrasse 1, 80336 Munich, Germany

**Keywords:** Early modeling, Decision analysis, Value-based pricing, Vascular closure devices

## Abstract

**Background:**

As economic considerations become more important in healthcare reimbursement, decisions about the further development of medical innovations need to take into account not only medical need and potential clinical effectiveness, but also cost-effectiveness. Already early in the innovation process economic evaluations can support decisions on development in specific indications or patient groups by anticipating future reimbursement and implementation decisions. One potential concept for early assessment is value-based pricing.

**Methods:**

The objective is to assess the feasibility of value-based pricing and product design for a hypothetical vascular closure device in the pre-clinical stage which aims at decreasing bleeding events. A deterministic decision-analytic model was developed to estimate the cost-effectiveness of established vascular closure devices from the perspective of the Statutory Health Insurance system. To identify early benchmarks for pricing and product design, three strategies of determining the product’s value are explored: 1) savings from complications avoided by the new device; 2) valuation of the avoided complications based on an assumed willingness-to-pay-threshold (the efficiency frontier approach); 3) value associated with modifying the care pathways within which the device would be applied.

**Results:**

Use of established vascular closure devices is dominated by manual compression. The hypothetical vascular closure device reduces overall complication rates at higher costs than manual compression. Maximum cost savings of only about €4 per catheterization could be realized by applying the hypothetical device. Extrapolation of an efficiency frontier is only possible for one subgroup where vascular closure devices are not a dominated strategy. Modifying care in terms of same-day discharge of patients treated with vascular closure devices could result in cost savings of €400-600 per catheterization.

**Conclusions:**

It was partially feasible to calculate value-based prices for the novel closure device which can be used to inform product design. However, modifying the care pathway may generate much more value from the payers’ perspective than modifying the device per se. Manufacturers should thus explore the feasibility of combining reimbursement of their product with arrangements that make same-day discharge attractive also for hospitals. Due to the early nature of the product, the results are afflicted with substantial uncertainty.

## Background

As economic considerations become more important in healthcare reimbursement, decisions about the further development of medical innovations need to take into account not only medical need and potential clinical effectiveness, but also the cost-effectiveness of a new drug or medical device. It has been proposed that already in an early phase of the innovation process, economic evaluations can support decisions on further development in specific indications or patient groups by anticipating future reimbursement and implementation decisions [1, 2]. Early assessment and estimation of health and economic outcomes are vital for making these decisions, and modeling techniques can be used to achieve this [3, 4].

One potential concept for early assessment is value-based pricing. It can be used to evaluate the additional value that can be achieved by adopting an innovative medical technology and to set a price relative to that value [5]. The question of what constitutes “value” of health technologies is a matter of unresolved debate (for Germany, see e.g. [6–8]). Following Sussex et al., value-based pricing requires an agreement about how the relevant benefits and costs are identified, measured, valued, aggregated, and used in decisions [9]. One central attribute of value of new health technologies is the health gain it provides. Value-based pricing is frequently assumed to involve comparing the incremental cost-effectiveness ratio of the novel technology with a threshold for cost-effectiveness defined by the decision maker [10]. Given that cost-effectiveness varies between patient subgroups, it is sensible for innovators to calculate a menu of prices to determine the patient group in which their intervention is most likely to be cost-effective [10, 11]. Furthermore, if used early in research and development (R&D), economic evidence might also be useful for value-based product design. Here, development activities are oriented towards attributes which are most valuable to those who decide about the innovations’ reimbursement and implementation in clinical practice.

The German Institute for Quality and Efficiency in Healthcare (“Institut für Qualität und Wirtschaftlichkeit im Gesundheitswesen”, IQWiG) proposes using an “efficiency frontier” based on the cost-effectiveness within a medical condition as a benchmark of the German Statutory Health Insurance (SHI) fund’s valuation of an innovation [12]. Traditionally, measuring “efficiency” of health services in Germany does not imply comparing a new treatment’s cost-effectiveness to any threshold value, but rather assessing whether one alternative is dominated. Thus, prices for which a new technology would be cost-saving or at which it incurs the same incremental cost per health outcome as the most effective existing technology, may serve as two benchmarks of value in the German context.

A recent systematic review by Markiewicz et al. [13] provides an overview of applied early assessment; there are, however, few case studies on early assessment in the context of the German SHI system available in the literature. This manuscript provides a case-study for value-based pricing and product design in the area of cardiovascular diseases which constitute a substantial part of the economic burden of disease in industrialized countries such as Germany.

The standard therapy for diagnosing and treating coronary diseases in Germany is cardiac catheterization via a femoral artery access. In 2011, around 1.2 million diagnostic and 600,000 interventional (incl. transcatheter aortic valve implantations) heart catheterizations were performed in German hospitals [14]. To reduce time to normalization of hemostasis and sheath removal, various vascular closure devices (VCDs) were developed as an alternative to commonly applied manual compression [15–19]. Over 330,000 VCDs were used in inpatient care in Germany in 2011. After diagnostic catheterization, about 30 % of patients received a VCD, after percutaneous coronary intervention (PCI) about 45 %, and after transaortic valve transplantation (TAVI) 56 %, respectively [20]. A number of health economic evaluations, identified in a qualitative review, analyze closure devices, with controversial results regarding cost-effectiveness. The overall notion is that VCDs reduce time to hemostasis, which may lead to earlier ambulation and cost-savings. However, severe complications and associated follow-up costs seem to increase with the use of VCDs [15, 21–27].

One area of medical innovation in this field is to develop novel VCDs, which prevent bleeding complications after sheath removal. Similar devices are currently developed by German researchers in cooperation with industry partners and sponsorship by the German Federal Ministry of Education and Research. The purpose of this study is to provide an example of using value-based pricing and product design in the early economic evaluation of an innovative VCD for cardiac diagnosis and interventions in which methods of regenerative medicine are used to reduce the number of bleeding complications. Given the early development stage of the new device, the aim was not to develop a complex, fully probabilistic model which would be required to provide economic evidence for a national coverage and reimbursement decision. Instead, the aim was to generate an early estimate of value from an Statutory Health Insurance (SHI) perspective to identify attractive target patient groups, inform tentative value-based prices for differing assumptions of effectiveness, and derive implications for including such value considerations into product design.

## Methods

### Model structure

**Fig. 1:**
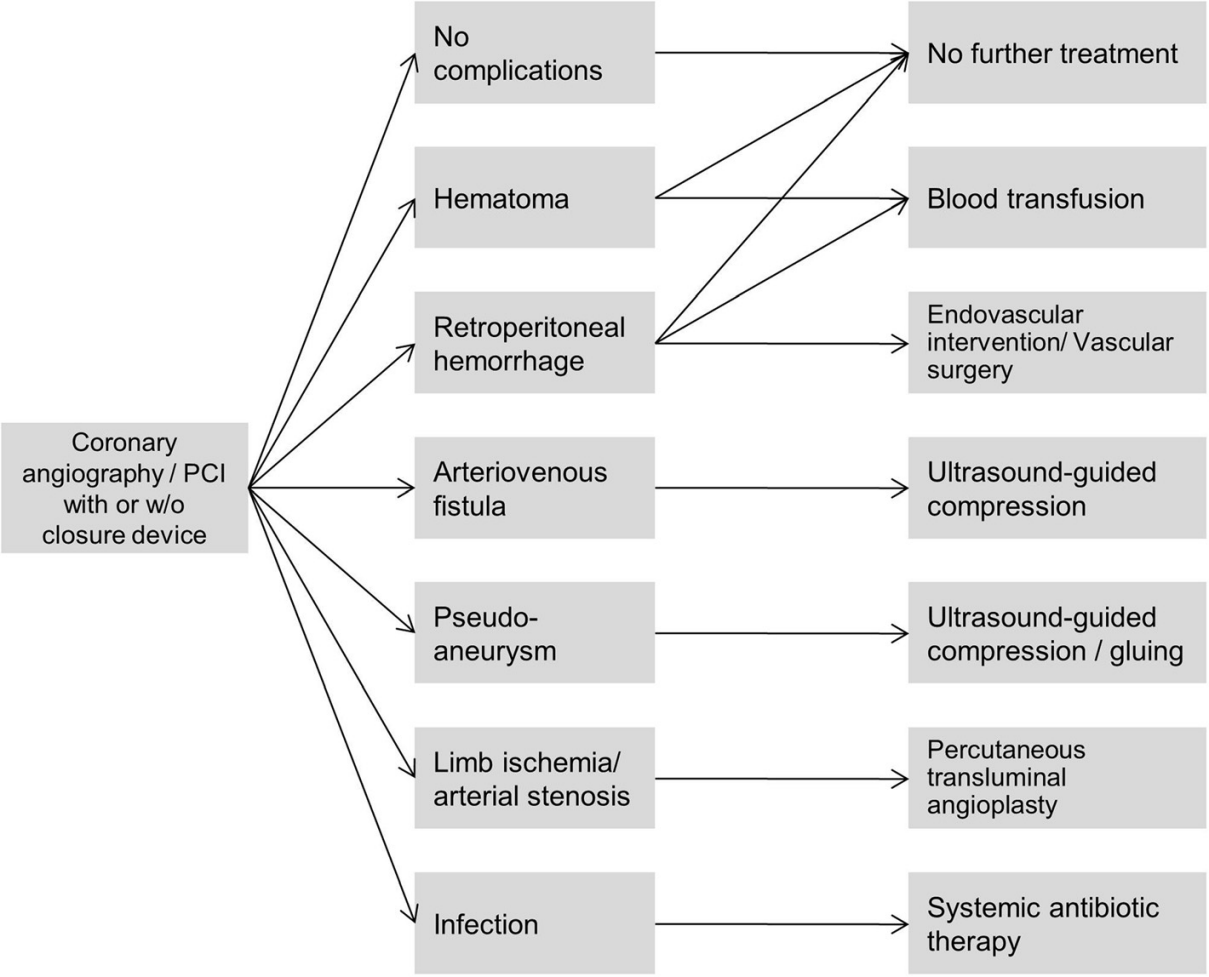
Decision tree. Not shown is the decision arm depicting a failure of the vascular closure device

A decision tree is used to depict relevant access site related complications following coronary angiography or percutaneous coronary interventions and subsequent therapies (Fig. 1). The alternative methods to achieve hemostasis after sheath removal are manual compression and application of VCDs. Manual compression (MC) is chosen as a comparator in accordance with existing economic evaluations, because this is the standard method to stop bleeding when smaller sheath sizes are used [15, 21–25, 27]. Interventions requiring large sheath sizes (>18 French), such as transcatheter aortic valve implantations, are not included in the model as hemostasis methods apart from VCD are not comparable (surgical arteriotomy vs. manual compression) [28, 29].

The comparator ‘vascular closure device’ represents the mean costs and effects of the variety of devices currently used in German inpatient care and serves as a benchmark for the novel device. The novel device is considered in the analysis as a hypothetical scenario: a new comparator for which the effectiveness is varied between that of existing VCDs and a VCD with 100 % effectiveness, i.e. zero bleeding complications. A time horizon of 24 h is chosen in accordance with published studies and expert opinion, which covers the time from sheath removal to cure of all complications and hospital discharge [22, 23, 30, 31].

Following German guidelines for health economic evaluation, this study aimed at assessing costs from a societal perspective [32]. Given that the majority of patients undergoing coronary catheterization is older than 65 years and can therefore assumed to be retired [33], indirect costs due to loss of productivity were considered less relevant. Also, no direct non-medical costs (particularly travel costs to point of care) were identified which differ across the strategies and equally no differences between direct medical costs not covered by the German SHI. Therefore, this study assesses costs from the health care system perspective. Only inpatient treatment of complications is relevant for this assessment as the majority of complications and costs incur at hospital.

Given the short time horizon and the acute nature of complications, the intervention is modeled as a decision tree ([34], p. 23). Effectiveness is measured by the total number of complications averted per catheterization in each respective case. All data in the model are point estimates. Discounting effects and costs is not necessary as they occur within 24 h. All calculations are carried out using MS Excel 2007.

The starting point of the model is immediately after sheath removal and either beginning of manual compression or insertion of a VCD. Patients either remain complication free or develop one or several of the following complications: hematoma, retroperitoneal hemorrhage, arteriovenous fistula, pseudoaneurysm, limb ischemia/arterial stenosis, and infection. These complications are compiled from the literature and are considered relevant for identifying the effectiveness and safety of closure devices [15–17, 19, 21, 24–26, 35]. Furthermore, complications are validated by clinical experts. In case of VCD, the decision tree contains an extra arm for the possibility of device failure [15, 16, 18, 23, 24, 26, 35]. It is assumed that manual compression is applied to achieve hemostasis after VCD failure [15, 16, 26]. Complications are modeled independently. We assume that there are no recurrences and that every complication - with the exception of minor hematomas and minor retroperitoneal hemorrhages - is treated immediately [15, 21, 26].

Effectiveness of VCDs compared to manual compression is measured as the number of averted complications per catheterization. This method has been used previously in meta-analyses and economic evaluations on closure devices [15, 18, 25, 26]. For a conservative estimate of effectiveness, only hematomas graded as large are included in the analysis, whereas hematomas graded as small and medium are not considered; it is assumed that large graded hematomas have a similarly severe impact on patients’ well-being as the other complications considered in the analysis [16]. In the VCD arm of the analysis, also the number of complications due to manual compression after failure of the device is considered.

### Clinical parameters

We identified five meta-analyses containing data on complications associated with manual compression and VCDs [15–19]. The most recent meta-analysis is chosen as data source because it contains the largest number of patients and trials and follows the methodological recommendations of the Cochrane Collaboration [16]. The study compares the safety and efficacy of various VCDs after transfemoral diagnostic or interventional angiography with manual compression. Thirty-one prospective, randomized trials from 1992 to 2008 with 7,528 patients are included. However, high-risk patients were excluded in most studies. Collagen-based and suture mediated devices are incorporated in the analysis, and sheath sizes range from 3 to 10 French. The chosen meta-analysis provides probabilities for all complications, except arteriovenous fistulae. Data are reported separately for diagnostic and interventional catheterization.

Data on the probability of developing an arteriovenous fistula are extracted from a pooled analysis of randomized trials on the use of AngioSeal after interventional catheterizations, which includes 10,113 patients [21]. Probabilities for treatment of hematomas with blood transfusion are gained from a meta-analysis of 27 studies with a total of 3,010 participating patients [15]. Manual compression after diagnostic or interventional catheterization is compared with the use of hemostasis devices (VasoSeal, Kensey Nash). Due to a lack of published data, the probability for treating retroperitoneal hematomas with blood transfusions, endovascular intervention (embolization or stent-graft), or vascular surgical intervention is based on a clinical expert’s opinion [36, 37]. To account for higher uncertainty, this parameter received special attention in the sensitivity analysis. It is assumed that all other complications are treated with one standard therapy, which was chosen in accordance with published literature and clinical experts’ opinions [15, 16, 26, 38–40]. Tables 1 and 2 provide an overview of clinical parameters used in the analysis.

**Table 1:** Complication rates

Diagnostic & Interventional	Hematoma	AVF	PSA	RPH	Limb Ischemia	Infection
VCD	4.56 %	0.83 %	1.60 %	3.79 %	0.31 %	0.61 %
MC	5.08 %	0.20 %	1.59 %	2.91 %	0.00 %	0.22 %
Source	(13)	(18)	(13)	(13)	(13)	(13)
Diagnostic	ᅟ	ᅟ	ᅟ	ᅟ	ᅟ	ᅟ
VCD	4.20 %	0.83 %	0.90 %	4.40 %	0.00 %	0.10 %
MC	5.70 %	0.20 %	0.00 %	5.30 %	0.00 %	0.10 %
Source	(13)	(18)	(13)	(13)	(13)	(13)
Interventional	ᅟ	ᅟ	ᅟ	ᅟ	ᅟ	ᅟ
VCD	4.40 %	0.83 %	2.60 %	3.60 %	0.30 %	0.90 %
MC	4.80 %	0.20 %	2.50 %	2.40 %	0.00 %	0.30 %
Source	(13)	(18)	(13)	(13)	(13)	(13)

Data on AVF are for interventional catheterization only

*AVF* arteriovenous fistula, *MC* manual compression, *PSA* pseudoaneurysm, *RPH* retroperitoneal hemorrhage, *VCD* vascular closure device

**Table 2:** Probabilities for treatment and failure of VCD

Treatment	Blood Transfusion (Hematoma)	Blood Transfusion (RPH)	Endovascular Intervention/ Vascular Surgery (RPH)	US-guided compression (AVF, PSA)	PTA (Limb ischemia)	Antibiotics (Infection)
VCD	0.20 %	1.00 %	1.00 %	100 %	100 %	100 %
MC	0.30 %	1.00 %	1.00 %	100 %	100 %	100 %
Source	(12)	Clinical expert	Clinical expert	Clinical expert	Clinical expert	Clinical expert
VCD failure	3.60 %		Source		(13)	

*AVF* arteriovenous fistula, *MC* manual compression, *PSA* pseudoaneurysm, *PTA* percutaneous transluminal angioplasty, *RPH* retroperitoneal hemorrhage, *US* ultrasound, *VCD* vascular closure device

### Costs

All costs in the model are obtained as reimbursement rates for diagnosis-related groups (DRGs) from the German Hospital Reimbursement Institute (“Institut für das Entgeltsystem im Krankenhaus”, InEK) [33]. DRGs are chosen because they best reflect the costs borne by statutory health insurance and are used regularly in the literature to calculate inpatient costs [40, 41].

We analyze the 2010 DRG statistic provided by InEK to identify DRGs that are associated with the procedure code for VCDs. In a second step, these DRGs are narrowed down by incidence of ICD-10 diagnosis codes for all relevant complications [42]. The identified DRGs are validated by the controlling department of a university hospital. Table 3 shows all groups, which are considered as best representatives of the costs incurred by complications. Basic DRGs F49G (diagnostic) and F24B/ F19C (interventional) are used for the reimbursement of catheterization without complications. Given that the basic DRGs are the same for manual compression and use of VCD, cost differences result from subsequent complications. Treatment of complications is either reimbursed by a distinct DRG (infection treatment, endovascular intervention/ vascular surgery) or covered by a higher weighted DRG of catheterization (F49A and F24A/ F19A). Costs attributable to complications are then calculated as the difference between the higher weighted and the lower weighted DRG of catheterization. The costs of VCDs per se are not separately reimbursed through the DRG-system. The reimbursement for achieving hemostasis is included in the basic DRG for diagnostic or interventional catheterization and is the same for all methods.

**Table 3:** G-DRG description

Rationale	DRG	Name
Basic DRGs		
Diagnostic w/o complications	F49G	Invasive cardiologic diagnostic without complications
Interventional w/o complications	F24B	Percutaneous coronary angioplasty without complications
	F19C	Percutaneous transluminal intervention at the heart without complications
Higher weighted DRGs (blood transfusion, ultrasound-guided compression, gluing, and percutaneous transluminal angioplasty)		
Diagnostic w/o complications	F49A	Invasive cardiologic diagnostic with major complications
Interventional w/o complications	F24A	Percutaneous coronary angioplasty with major complications
	F19A	Percutaneous transluminal intervention with major complications
Distinct DRGs for complications		
Treatment of infection	T61B	Postoperative infection
Surgery for vascular complications	F08E	Reconstructive vascular intervention without complications

*G-DRG* German diagnosis-related group

The costs for diagnostic and interventional catheterizations combined are calculated as a weighted average (1:2), which was derived from the proportion of diagnostic and interventional catheterizations enclosed in the meta-analysis by Biancari et al. [16]. All costs are reported in Euros (€) of the financial year 2010 and rounded to the nearest euro. Table 4 shows all relevant cost data.

**Table 4:** Cost data

Treatment	Type of catheterization	Total costs (€)		Cost
		With complications	W/o complications	Difference (€)
Blood transfusion/PTA	Diagnostic	F49A: 6.385,32	F49G: 1,085.33	5,299.99
	Interventional	F24A: 7.741,65	F24B: 5.108,25	2,633.40
	Diagnostic & interventional			3,522.26
US-guided compression/gluing	Diagnostic	F49A: 6.385,32	F49G: 1,085.33	5,299.99
	Interventional	F19A: 7.063,48	F19C: 4.603,30	2,460.18
	Diagnostic & interventional			3,406.78
Endovascular int./Vascular surgery	Diagnostic, interventional, diagnostic & interventional	F08E: 6,834.49 €		-
Systemic antibiotic treatment	Diagnostic, interventional, diagnostic & interventional	T61B: 1,735.05 €		-

Costs for the diagnostic & interventional group are a weighted average of diagnostic and interventional costs (1:2)

*G-DRG* German diagnosis-related group, *int.* intervention, *PTA* percutaneous transluminal angioplasty, *US* ultrasound

### Analysis

The costs and effects of existing VCDs compared to manual compression are calculated for a combination of diagnostic and interventional catheterizations (base case). To explore which patient populations are suitable target groups for the novel device, costs and effects are calculated separately for diagnostic and interventional catheterizations. Scenario-analysis is conducted because of evidence that larger sheath sizes used for interventional compared to diagnostic catheterizations result in higher complication rates [16]. Furthermore, use of closure devices is recommended primarily after insertion of large sheath sizes beginning at 7 French. According to the 2011 statistics of DRG reimbursement in Germany, about 30 % of patients receive a VCD after diagnostic catheterization and 45 % after interventional catheterization [20].

To assess the potential value of different specifications for the innovative device in terms of reduced costs attributable to complications, we change the effectiveness of vascular closure devices in a scenario-analysis (ceteris paribus). As the novel device is in the pre-clinical stage, only assumptions can be made regarding its effectiveness in preventing complications in accordance with the manufacturer. The novel device is supposed to decrease complication rates of hematomas and retroperitoneal hemorrhages (bleeding events); thus, we decrease rates in steps of 10 % until all bleeding complications are averted. Potential cost savings due to higher effectiveness in preventing bleeding events are calculated as the difference in costs attributable to complications of established VCDs and the novel device. In addition, we change all other complication rates to investigate which complications have the strongest impact on costs. We conduct a deterministic sensitivity analysis of the base case model to assess the impact of each effect parameter, including the probability of having no complications, on costs. To achieve this, each effect parameter is varied separately in the range of the upper and lower 95 % confidence limit as reported by Biancari et al. [16]. The range of costs is plotted in form of a tornado chart.

The German IQWiG recommends using the cost-effectiveness of current care as a benchmark of willingness to pay for a health gain [43]. To assess the potential value of a device designed to prevent bleeding complications in terms of willingness to pay for a health gain, we estimate the incremental cost effectiveness ratios (ICERs) of using closure devices compared with manual compression as far as possible for the different target groups. Given that no consistent estimates of health-related quality of life were identified for the different complications and given that the IQWiG does not specify methods of aggregating and valuing different endpoints, the ICERs were calculated for the number of averted complications per catheterization.

Finally, we assess a potential further way how the use of the novel device could be valuable for the SHI. Because of evidence that cost savings due to same-day discharge of patients treated with VCDs after diagnostic catheterization are possible [22, 23], we estimate potential savings for the German context in a secondary analysis. From the DRG for diagnostic catheterization used in our model (F49G), the reduction of resource costs is calculated by extracting parameters reflecting costs possibly avoided by earlier discharge (ward, intensive care unit) [44]. Alternatively, reimbursement savings are calculated as the difference between inpatient (DRG F49G) and outpatient (doctors’ fee scale: positions 13542, 01520, 34291, 40300) reimbursement for diagnostic catheterization [45].

## Results

### Costs and effects of existing VCDs

In the base-case analysis, the manual compression strategy yields total costs per catheterization of €3,667. Of these costs, €68 are due to the 0.10 complications per catheterization. The costs of the VCD strategy sum up to €3,706 per catheterization, whereas €107 of these costs are associated with 0.12 complications per catheterization.

The scenario analysis of diagnostic catheterization results in incremental costs of €77 and a reduction of 0.0084 complications per catheterization for the VCD strategy when compared to manual compression. The interventional catheterization scenario shows additional €36 and additional 0.023 complications per catheterization in the VCD group. Table 5 provides an overview of absolute and incremental costs and effects. Positive values of incremental cost effectiveness mean that under MC complications are prevented compared to VCD; a positive signal thus represents a more favorable outcome of MC compared to VCD.

**Table 5:** Total and incremental effectiveness and costs

Type of catheterization	Method of hemostasis	Total number of complications (per catheterization)	Total costs (€/catheterization)	Incremental effectiveness (# complications prev./ catheterization)	Incremental costs (€/catheterization)	ICER (€/complication prev.)
Diagnostic & interventional	MC	0.100	3,667	0.016	39	Dominated
	VCD	0.116	3,706			
Diagnostic	MC	0.113	1,105	−0.008	77	9,142
	VCD	0.105	1,182			
Interventional	MC	0.102	4,930	0.023	36	Dominated
	VCD	0.125	4,966			

*ICER* incremental cost effectiveness ratio, *prev.* prevented

### Potential savings from avoided complications

**Fig. 2:**
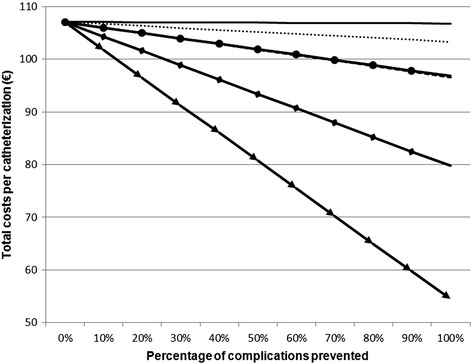
Change in total complication costs per catheterization by change in effectiveness (base-case). Hematoma.  Retroperitoneal Hemorrhage.  Arteriovenous Fistula.  Pseudoaneurysm.  Limb ischemia/arterial stenosis.  Infection

Figure 2 illustrates the effect of improving the effectiveness of VCDs in terms of averted complications. For the base-case, a 100 % prevention of hematomas and retroperitoneal hemorrhages has the lowest effect on costs resulting from complications. Cost savings for hematomas and retroperitoneal hemorrhages are €4 per catheterization in total. Preventing all pseudoaneurysms would have the largest potential for savings with an amount of €53 per catheterization (Table 6).

**Table 6:** Cost savings by complication

Complication 100 % averted	Cost savings per catheterization (€)
Hematoma	1
Retroperitoneal hemorrhage	4
Limb ischemia/ arterial stenosis	11
Infection	10
Arteriovenous fistula	27
Pseudoaneurysm	53

To assess whether cost savings from hematoma and retroperitoneal hemorrhage depend on the type of catheterization, complication rates are also varied in diagnostic and interventional scenarios. The result is similar to base-case analysis with total savings of €6 and €4, respectively.

**Fig. 3:**
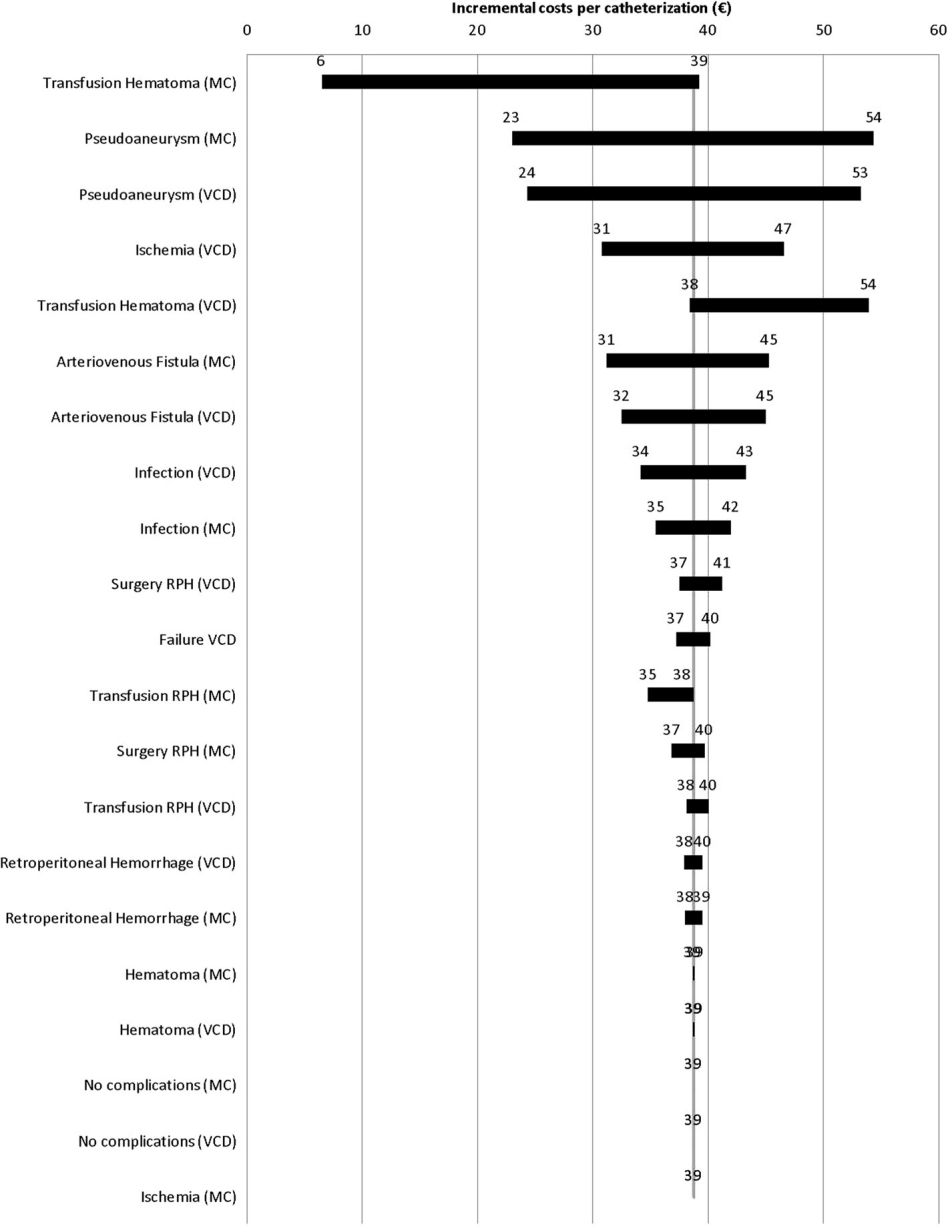
Tornado chart. MC, manual compression; RPH, retroperitoneal hemorrhage; VCD, vascular closure device

The deterministic sensitivity analysis of parameter variations within their 95 % confidence intervals shows that the probability for blood transfusion in case of hematoma in the manual compression strategy has the largest effect on incremental costs. Results also are especially sensitive for the probability of developing a pseudoaneurysm in both strategies. Variables estimated by a clinical expert apparently do not considerably influence incremental costs. Figure 3 shows results of the sensitivity analysis.

### Potential willingness to pay for avoided complications

Both for the base-case and for interventional procedures, established VCDs incur higher costs and more complications compared to MC so that they are a dominated strategy. It is therefore not possible to use the IQWiG-benchmark for valuing novel drugs [43] and to extrapolate the ICER of the most effective current treatment as a benchmark for an acceptable cost-effectiveness ratio for a novel device.

If closure devices are used after diagnostic catheterization, they are associated with fewer complications and higher costs. This is due to the fact that the complications they are associated with are more costly than those of MC. Assuming that all complications are similarly severe from a medical and patient perspective so that effectiveness can be measured by the number of complications avoided, an ICER could be calculated which amounts to €9,142 per averted complication.

As specified by the manufacturer, the new device is designed to prevent bleeding complications. Comparing a hypothetical device with neither hematoma nor retroperitoneal hemorrhage with manual compression results in a lower number of complications and higher costs for all three scenarios (base case: 35€/-0,064 complications/ICER €547 per averted complication, diagnostic: 71€/-0,091 complications/ICER €780 per averted complication, interventional: 32€/-0,053 complications/ICER €604 per averted complication) [43].

### Potential value of modifying the pathway of care

The novel hypothetical device is assumed to minimize bleeding complications, especially after interventions with large sheaths, which makes same-day discharge safer than with existing VCDs. In Germany however, patients undergoing interventional catheterization have to stay at hospital for observation for at least 24 h, regardless of the method used for achieving hemostasis. In theory, same-day discharge is possible for patients treated with a VCD after diagnostic catheterization; however, this approach does not seem to be common German practice, as inpatient stay is covered by DRGs for diagnostic catheterization. In 2011, just about 2.6 % of diagnostic catheterizations coded in DRG F49G were carried out on a day care basis [46].

One potential indicator of the potential value of modifying the care pathway towards same-day discharge can be found within the DRGs for interventional and diagnostic procedures which include cost for hotel and general wards at an amount of approximately €400 per patient. These costs could be avoided by same-day discharge. A second point of orientation for potential savings from same-day discharge can be taken from ambulatory (rather than in-patient) reimbursement for the procedures which is about 600€ lower than the DRG reimbursement.

## Discussion

### Using the analysis for value-based pricing and product design

By increasing the effectiveness of closure devices, cost savings are possible. Price negotiations with health insurance companies could be based on the aim to transfer these into reimbursement rewards for manufacturers, for example, in the form of premiums on specific DRGs for coronary catheterization. These premiums could represent a price up to which the use of the hypothetical device is a cost-saving strategy compared to the standard approach; statutory health insurers would not be at a financial disadvantage but rather offer additional benefit to patients and doctors.

In this analysis, only cost savings of €4 per catheterization could be obtained if the novel device was designed in a way that 100 % of bleeding complications could be avoided. Applying value-based pricing would lead to a premium of up to only €4. Changing the R&D strategy towards designing a product that would be associated with a reduction of other complications than bleeding (such as pseudoaneurysms and arteriovenous fistulae) might be desirable as cost savings are higher there. For example, preventing all pseudoaneurysms would increase the value-based price to €53, which is still a modest price markup.

A second possible way to deduce a value-based price is to extrapolate the efficiency frontier as proposed by the German IQWiG. Assuming a willingness-to-pay as high as the current treatment standard, a premium of up to €835 per diagnostic catheterization might be realized in reimbursement negotiations. This would represent a price where the use of the hypothetical device is still a cost-effective strategy compared to established products. It is unclear, though, whether the willingness-to-pay for additional effectiveness of health insurance funds corresponds with the IQWiG concept. Using willingness-to-pay thresholds is more likely to be relevant for product design in the case of decision makers with more explicitly stated values and methods like the National Institute of Health and Clinical Excellence in the UK.

A third way to use this analysis is to identify reimbursement or care scenarios in which the novel device would be valuable from a SHI perspective. The study demonstrated that considerable cost savings (€400-600 per patient) are possible if patients are discharged on the day of catheterization. However, it is likely that from a hospital perspective the decrease of reimbursement revenues is higher than the resource savings due to earlier discharge; hospitals are likely to have financial incentives not to discharge patients on the day of catheterization. To account for these results, the value-based pricing strategy could be extended beyond the scope of the device per se to modify care pathways.

Such an approach is only likely to be acceptable to clinicians and patients if complications can be reduced to an amount that it is considered safe to discharge patients immediately. The meta-analysis identified during this study revealed that the application of VCDs increases the risk for severe complications - especially limb ischemia/arterial stenosis and infection. To ensure that the novel device is reimbursed and implemented in clinical practice, these complications should be accounted for in the product development process.

### Comparison of results with existing evidence

The cost studies by Mann et al. [26] and Reddy et al. [24] report higher costs for the VCD strategy compared to manual compression, as in our study. However, differences in cost result in costs of the medical device itself, which are relevant from the hospital’s perspective. Costs of the device itself cannot be assessed in this analysis, as medical devices for hemostasis are not reimbursed on a fee for service basis in the German DRG-system. Similar results, however, would be expected, when employing reimbursement premiums for the hypothetical device. Studies assuming same-day discharge of most patients in the VCD group conclude that using a device is a cost-saving strategy [22, 23]. We do not include early discharge as a structural assumption in the model. Secondary analysis, however, shows considerable potential cost savings. If manual compression leads to significantly more complications than application of VCDs, the closure device strategy is cost-saving as well [21].

We identified one other study where an incremental analysis of costs and consequences is conducted. Similar to the results of this analysis, Bos et al. [15] calculate an ICER of $9,000 per averted complication for VCDs versus manual compression after diagnostic and interventional femoral catheterization.

### Strengths and limitations

To our knowledge, this is the first study evaluating the feasibility of calculating value-based prices and providing recommendations for value-based product design for innovative closure devices.

This study only included direct medical costs of inpatient care on the basis of DRGs which provide an estimate of the average costs assessed with the VCD’s complications. However, decisions about the acquisition of new closure devices are typically made by hospitals within the context of the DRG system. This study already addressed the role of potentially adverse incentives implied by the DRG system. Further research should explore the value from a hospital perspective because on this level.

The structure of the model is coherent with treatment pathways in Germany and is validated by several clinical experts. A time horizon of 24 h is chosen, which is appropriate to reveal costs and consequences relevant for inpatient analysis. Costs of the whole hospital episode are considered in the analysis, which might also include hospital stay longer than 24 h. To analyze costs and consequences in outpatient care - for example work absenteeism due to complications - and consider long-term effects of either manual compression or application of VCDs, a longer time horizon would be required. Use of closure devices in interventions with large sheath sizes as used for transcatheter aortic valve implantations, could not be considered as a subgroup analysis in the model. Here, the alternative method to achieve hemostasis compared to use of VCDs is surgical cutdown [29, 47, 48]. The benefit of using VCDs instead of open surgery in transcatheter aortic valve implantations is difficult to estimate as there is evidence on lower rates of vascular complications and higher rates of bleeding events [29, 48].

Assumptions on the value of the outcome measure needed to be made to calculate value-based prices. It was assumed that patients value fewer complications higher than more complications and that interval property is given [12]. It is also assumed that the different complications aggregated in the outcome measure have a similar impact on patients’ wellbeing, which might not be the case in clinical practice. The value for patients might extend beyond this outcome measure. Patients are reported to appraise methods of achieving hemostasis, for example, regarding their ability to eat and difficulty to urinate after catheterization [35, 49]. Further work is necessary to assess the patients’ relative valuation of the different outcomes and their willingness to pay out of pocket for these aspects. Due to the lack of published generic measures of quality of life after either manual compression or insertion of a vascular closure, no cost-utility analysis was developed. Also, the analysis focuses on the German context where the willingness-to-pay threshold currently proposed by IQWiG is not based on quality-adjusted life years by default [12].

The efficiency frontier approach used in this analysis has met substantial criticism. It has been argued that there is no scientific or normative rationale for using the cost-effectiveness of the current treatment standard as a valid measure of willingness-to-pay. Addressing this criticism is beyond the scope of this study. A detailed discussion can be found in the statement of several German health economists [50, 51] and by Drummond and Rutten [52]. Further limitations of the IQWiG approach become apparent in our analysis, as the efficiency frontier can only be used to estimate the cost-effectiveness of a subgroup of catheterizations; no recommendation on value-based prices can be given for base case and interventional scenarios. The efficiency frontier approach is applied nevertheless for this early evaluation, because the analysis focuses on value-based pricing in the German SHI system and no alternative benchmark for Germany is currently available.

Further research could be conducted to refine the model regarding the following issues: Data on complication rates are extracted from three meta-analyses. This might distort results as different closure devices and types of catheterization are included. Furthermore, the data used in this early model are not sufficient to provide a detailed analysis of all VCDs currently used in Germany. This was considered a reasonable starting point because decisions about reimbursement rates in Germany are typically oriented at methods in general rather than specific products used within them. However, it would be desirable to extend this work and include data on all comparators, in particular the most effective one. Also, the meta-analyses include data from studies conducted over 20 years ago. Treatment practice and experience in handling VCDs have evolved since then, which might possibly lead to a higher effectiveness of VCDs in the present than represented in the data. Data regarding death as a complication of vascular closure device use could not be identified. Also, death could not be included in the chosen outcome measure ‘number of averted complications’ as the impact of death on patient’s health is not comparable to the impact of the other complications considered in the analysis. In general, the mortality rate is approximately 0.1 % after diagnostic catheterization and about 1 % after interventional catheterization [53–55]. The exclusion of death might overestimate the effectiveness of closure devices given that severe complications leading to death are more common with this method. Also, no data on high-risk patients with several co-morbidities or anticoagulation therapy are available because these are typically excluded from clinical trials. It is difficult to estimate in which direction results are distorted, as it is expected that patients with severely calcified arteries are better treated with manual compression. For very obese patients application of VCDs might be more effective [16]. Assuming the biggest possible price difference between DRGs with and without complications might overestimate costs attributable to complications. Furthermore, assuming that each patient with an arteriovenous fistula, pseudoaneurysm, limb ischemia, or infection is treated might lead to overestimation of the costs attributable to complications.

Also, a probabilistic instead of a deterministic model could have been developed. It is the purpose of this study to evaluate, how information on costs and effectiveness can be made usable to the manufacturer within a short period of research. Construction of a probabilistic model would be more time-consuming and beyond the scope of this project. Also, structural uncertainty is expected to be so high that parameter uncertainty is not as relevant as in other analyses. Also, it is unlikely that large efforts for refining the model would have changed the key finding that the potential savings from same-day discharge are much higher than those from a reduced number of complications.

## Conclusions

Generally it appears feasible to develop recommendations for value-based prices and product design strategies for novel closure devices on the basis of early health economic modeling. In the process of early evaluation, care pathways are identified and a potential for cost-savings due to value-based modifications becomes apparent: If the safety of discharging patients on the day of catheterization can be established for the hypothetical device, manufacturers should consider negotiating reimbursement arrangements based on the benefits from savings they incur. Cost-savings due to early discharge are more likely to provide an economic argument for using closure devices than savings due to prevented bleeding complications. Apart from the SHI perspective, also the willingness of patients to privately pay for greater comfort after interventions might be worth assessing by manufacturers. Further research is necessary to further explore the contribution of early economic evaluation to a value-based modification of care pathways and the role of other factors in decision practice.

## Abbreviations

DRG: Diagnosis-related group
ICER: Incremental cost effectiveness ratio
InEK: Institut für das Entgeltsystem im Krankenhaus, German Hospital Reimbursement Institute
IQWiG: Institut für Qualität und Wirtschaftlichkeit im Gesundheitswesen, Institute for Quality and Efficiency in Health Care
MC: Manual compression
PCI: Percutaneous coronary intervention
R&D: Research and development
SHI: Statutory Health Insurance
TAVI: Transaortic valve transplantation
VCD: Vascular closure device
